# Dual mobility total hip arthroplasty *vs*. bipolar hemiarthroplasty in treating patients with displaced femoral neck fractures: a systematic review and meta-analysis

**DOI:** 10.7717/peerj.21535

**Published:** 2026-07-16

**Authors:** Weichao Wang, Ziang Cheng, Keteng Xu, Fuqing Lin, Qing Yan

**Affiliations:** 1 Department of Joint Surgery, Huangshan City People’s Hospital, Huangshan, Anhui, China; 2 Clinical Medical College, Yangzhou University, Yangzhou, China; 3 Nanjing Luhe People’s Hospital, Yangzhou University, Nanjing, China

**Keywords:** Displaced femoral neck fractures, Dual mobility cup, Hip arthroplasty, Meta-analysis

## Abstract

**Background:**

Total hip arthroplasty (THA) has been reported with superior outcomes in hip pain, reoperation rate, function, but higher rate of dislocation compared with bipolar hemiarthroplasty (BHA) for treatment of displaced femoral neck fractures (DFNFs). A dual mobility cup (DMC) has been designed to reduce the rates of dislocation and re-operation. However, whether DMC-THA achieves better clinical outcomes than BHA remains ambiguous. Therefore, we have conducted this meta-analysis to investigate the clinical outcomes of DMC-THA *vs*. BHA in the treatment of DFNFs.

**Method:**

We identified studies in the following electronic databases, Medline, Embase, the Cochrane Library, and Web of Science before December 2025. The Preferred Reporting Items for Systematic reviews and Meta-Analysis (PRISMA) guidelines were strictly followed. We performed our research using Review Manager 5.4 software. This study was prospectively registered in INPLASY (registration number: INPLASY202040085; DOI: 10.37766/inplasy2020.4.0085).

**Results:**

Sixteen studies were eligible for the present meta-analysis, which enrolled 1,296 patients undergoing DMC-THA and 1,208 patients undergoing BHA. Compared with patients undergoing BHA, those undergoing DMC-THA showed significantly lower rates of dislocation (*P* < 0.00001), reoperation (*P* = 0.0004), one-year mortality (*P* < 0.0001), and last follow-up mortality (*P* = 0.01), along with higher Harris Hip Scores (HHS) (*P* = 0.001). However, the DMC-THA group ha d a longer operative time (*P* < 0.00001). No statistical differences were found in 1-month mortality (*P* = 0.29), 3-month mortality (*P* = 0.09), transfusion rate (*P* = 0.68), infection rate (*P* = 0.71), fracture (*P* = 0.81), loosening of components (*P* = 0.46), thromboembolism (*P* = 0.10), heterotopic ossification (*P* = 0.06), and hospitalization period (*P* = 0.22) between DMC-THA and BHA.

**Conclusions:**

DMC-THA may be associated with lower rates of dislocation, reoperation, and mortality compared with BHA in the treatment of DFNFs. However, given the predominantly observational nature of the included studies, these findings do not establish a definitive causal relationship. While DMC-THA is associated with a longer operative time, it does not appear to increase the risk of complications such as transfusion, infection, fracture, or thromboembolism. However, the evidence supporting these findings remains limited, and DMC-THA should be considered a potential treatment option rather than a routinely recommended approach for DFNFs. More high-quality studies with large sample sizes, adequate follow-up periods, and standardized outcome reporting are needed to further validate the long-term efficacy and safety of DMC-THA relative to BHA.

## Background

The rising volume of total hip arthroplasties (THA) is emerging as a significant factor in hip fracture epidemiology, as recent modeling data indicates that the increasing prevalence of hip replacements has mitigated the overall incidence by approximately 10.16%—replacing hips no longer at risk of fracture (Moldovan & Moldovan, 2025). Surgical treatments for displaced femoral neck fractures (DFNFs) aim to acquire early mobilization, decreased complications, and clinical improvement (Pala et al., 2024; Kim et al., 2018). However, the optimal treatment for DFNFs remains controversial (Bensen, Jakobsen & Krarup, 2014). Arthroplasty is a generally accepted treatment for elderly patients with DFNFs (Edelstein et al., 2023). Both bipolar hemiarthroplasty (BHA) and THA are effective treatments that enable early mobilization (Grammatikopoulos et al., 2025). BHA is a recommended treatment due to its quicker and relatively simpler procedure compared to THA; nearly 45% of elderly patients with DFNFs undergo BHA (Moldovan & Moldovan, 2025; Edelstein et al., 2023).

Compared with BHA, THA has been reported to have superior outcomes in hip pain, reoperation rate, postoperative function (Pala et al., 2024). However, THA has been reported with longer surgery time, more perioperative blood loss, and higher dislocation rate, which limit the use of THA for DFNFs to some extent. In order to reduce the dislocation rate, the dual mobility concept in THA was developed by Professor Gilles Bousquet in 1974 (Valcarenghi et al., 2022). This concept is composed of a metallic acetabular shell and a mobile polyethylene liner that allow for a larger prosthetic femoral head, which increases the range of motion and decreases the dislocation rate (Edelstein et al., 2023; Schulz et al., 2024; Chen et al., 2024). Although DMC-THA has been reported to be associated with a lower dislocation rate than conventional THA in the treatment of DFNFs (Zakaria et al., 2023; Ochi et al., 2017), several studies still focus on key clinical and economic differences when comparing BHA and DMC-THA in this patient population—specifically, the increased operative time, blood loss, hospital stay duration, and upfront costs associated with DMC-THA. There is no consensus on the recommended treatment.

Given the growing body of relevant studies published in recent years, an updated meta-analysis is warranted to inform clinical decision-making. Earlier systematic reviews and meta-analyses are now outdated, as they failed to incorporate high-quality studies published between 2022 and 2025 (Ma et al., 2021; Cha et al., 2020; Mufarrih et al., 2021; Liao et al., 2012; Yu, Wang & Chen, 2012). Moreover, recent efforts remain incomplete: Cicio et al. (2025) provided a qualitative systematic review in the absence of pooled effect estimates, limiting the statistical power of their conclusions. Similarly, Grammatikopoulos et al. (2025) lacked a comprehensive evaluation of critical safety outcomes. Specifically, their study did not quantitatively assess periprosthetic fractures, heterotopic ossification, infection, transfusion requirements, or length of hospital stay—endpoints that are essential for informed surgical decision-making.

Against this backdrop, there is a critical need to synthesize and evaluate evidence related to DMC-THA and BHA to identify the more effective treatment strategy for displaced femoral neck fractures (DFNFs). Therefore, the purpose of the present meta-analysis is to investigate the key clinical and safety outcomes of DMC-THA *vs*. BHA in the treatment of DFNFs.

## Methods

The research protocol for this review was determined by all coauthors before the literature searches began, and the study protocol was published online at the INPLASY (https://www.doi.org) under registration number INPLASY202040085 (DOI number is 10.37766/inplasy2020.4.0085). An update was published on October 22, 2025, extending the search period to December 2025 and incorporating methodological refinements; all amendments are documented in the INPLASY audit trail. This study strictly followed the Preferred Reporting Items for Systematic Reviews and Meta-Analysis (PRISMA) guidelines, with methodological design referencing frameworks from previous orthopedic meta-analyses.

### Inclusion and exclusion criteria

Studies meeting the following inclusion criteria were included: (1) Studies comparing the clinical outcomes of DMC-THA *vs*. BHA for DFNFs; (2) the studies providing extractable data; (3) studies published in English.

Studies were excluded for the following reasons: (1) Conference abstracts, reviews, case reports, sawbone or cadaver studies, studies relying solely on administrative database or registry data without clinical validation (*e.g*., insurance claims, national audits, propensity-matched cohorts derived from billing codes) because outcomes based on International Classification of Diseases (ICD) coding may misclassify complications and introduce bias, and non-comparable studies; (2) studies with insufficient data to extract key outcomes; (3) duplicate publications.

### Search strategy

We searched Medline, Embase, the Cochrane Library, and Web of Science from inception to December 2025 to retrieve relevant studies. No restrictions were imposed on publication year or study type to ensure comprehensiveness. The following key terms were used: “dual-mobility”, “dual mobility”, “double-mobility”, “double mobility”, “dual mobility cup”, “double mobility cup”, “DMC”, “Hemiarthroplasty”, “bipolar hemiarthroplasty”, “HA”, and “Hemi Arthroplasty”. This search strategy was adapted from frameworks used in meta-analyses of orthopedic interventions, which emphasize targeted terminology to capture all relevant literature.

### Data extraction and outcome measures

Two investigators (Weichao Wang and Ziang Cheng) independently extracted the relevant data using a standardized form. All disagreements were discussed until consensus was reached; if consensus could not be achieved, the third referee (Fuqing Lin) was consulted to resolve the dispute. The extracted information included two categories: (1) Basic study characteristics: first author, year of publication, country, study type, patient age, gender, number of DFNF cases, surgical approach, follow-up duration, and type of implants; (2) clinical outcomes at the final follow-up. Our selected outcomes were: (1) Dislocation rate; (2) reoperation rate; (3) mortality; (4) length of surgery; (5) intraoperative blood loss (milliliters, mL); (6) transfusion rate; (7) infection rate; (8) postoperative fracture; (9) loosening of Components; (10) thromboembolism (including deep vein thrombosis (DVT) and pulmonary embolism [PE]); (11) heterotopic ossification; (12) Hip Harris Score (HHS); (13) hospitalization period. The selection of these outcomes drew on the indicator logic of prior orthopedic meta-analyses, which prioritize clinically meaningful and patient-relevant endpoints.

### Quality assessment

For non-randomized controlled trials (non-RCTs), we used the Downs and Black scale and the Newcastle-Ottawa Scale (NOS), reflecting the distinct methodological requirements (Stang, 2010; Downs & Black, 1998). A score >13 on the Downs and Black scale (total: 0–28) indicated good or excellent quality. This dual-tool approach ensures optimal sensitivity for detecting bias specific to each non-RCT. The NOS has a total score of 9; studies with a score >6 were considered high-quality, as higher scores indicate more robust methodological design. For RCTs, a 12-item scale was used, with each item rated as “Yes,” “Unclear,” or “No” (Furlan et al., 2009). RCTs with >7 “Yes” responses were classified as high-quality, 4–7 “Yes” responses as moderate-quality, and ≤4 “Yes” responses as low-quality. Any discrepancies in quality assessment were resolved by a third reviewer. The inter-reviewer agreement was excellent, with a weighted kappa coefficient of 0.78—consistent with standards for reliable quality evaluation in orthopedic meta-analyses.

### Statistical analysis

Statistical analyses were performed using Review Manager 5.4 software. Heterogeneity was assessed using Cochran’s Q test (*P*-value reported) and quantified by the I²statistic. Heterogeneity levels were defined as: low (I^2^ ≤ 50%), moderate (I^2^ = 51–75%), and substantial (I^2^ > 75%). A fixed-effects model was used when heterogeneity was low; a random-effects model was used when heterogeneity was moderate or substantial. If I^2^ > 90%, no meta-analysis was performed for that outcome due to excessive heterogeneity.

For continuous data, the mean difference (MD) or standardized mean difference (SMD) with 95% confidence intervals (CIs) was used: MD was adopted when outcome measures across studies used the same unit, while SMD was applied when units were inconsistent. For dichotomous data, the odds ratio (OR) was calculated using the Mantel-Haenszel method. Statistical significance was set at *P* < 0.05.

Sensitivity analyses were conducted to evaluate the stability of results by sequentially excluding each eligible study. Publication bias for key outcomes (dislocation rate and reoperation rate) was assessed *via* visual inspection of funnel plots: a symmetric distribution indicated no significant publication bias, while asymmetry suggested potential bias. This analytical approach aligns with statistical protocols established in prior orthopedic meta-analyses, ensuring consistency and rigor.

## Results

### Study selection

**Figure 1:**
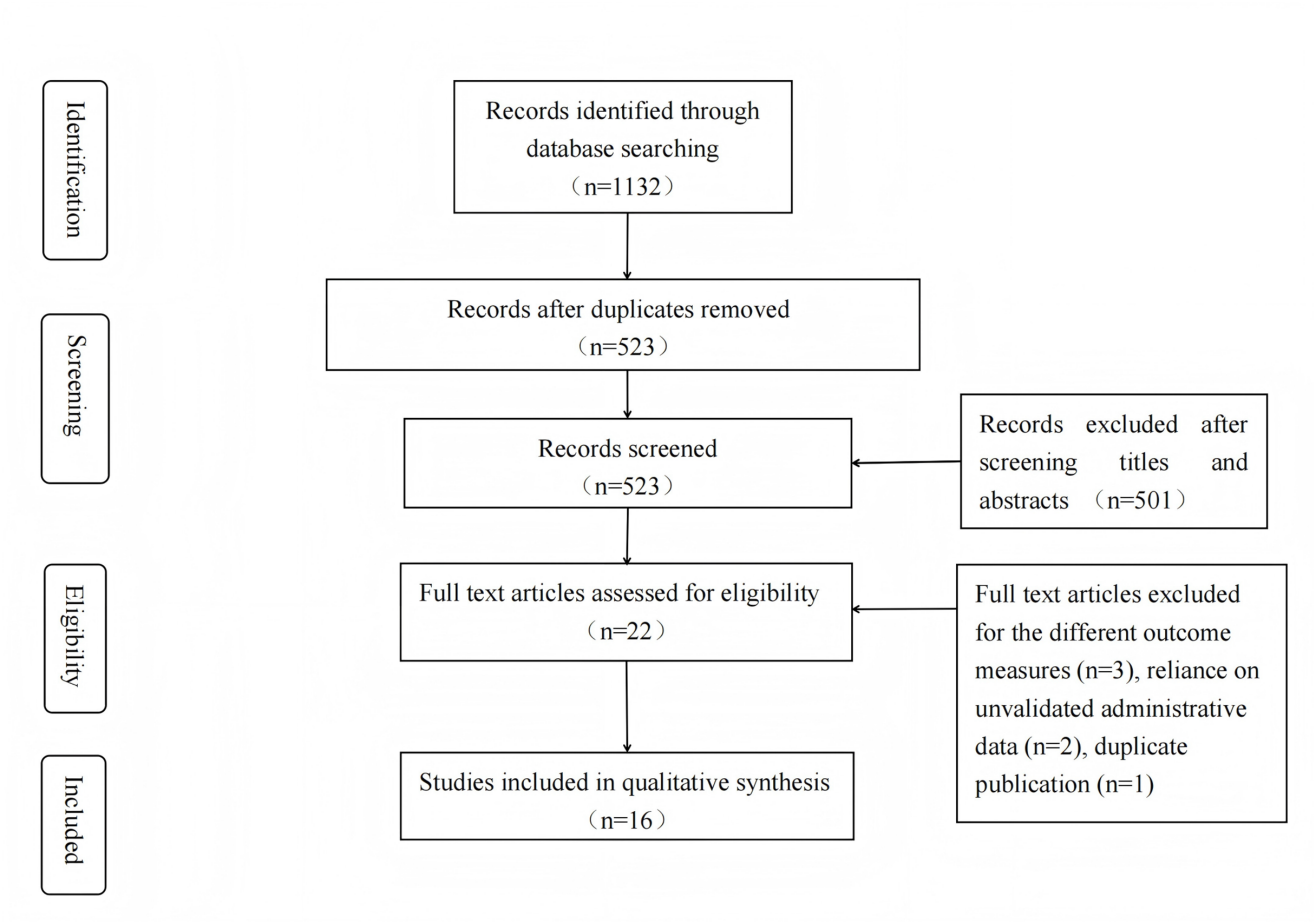
Flow chart of the study selection procedure.

By scanning the titles and abstracts, 22 articles that met the inclusion criteria were reviewed for full-text screening. After full texts were assessed for eligibility, some articles were rejected because they reported with insufficient data (Huten et al., 2019; Schmidt et al., 2020; Perrin et al., 2018), database analyses (Edelstein et al., 2023; Hong & Han, 2025), duplicate publication (Heshmat et al., 2024). Finally, 16 studies were eligible. The characteristics of the included studies in this meta-analysis are presented in Table 1. Regarding surgical approaches: 12 studies focused on the posterolateral approach and two studies on the direct anterior approach (Zakaria et al., 2023; Ochi et al., 2017). One additional study included three approaches (the anterior, anterolateral, and posterolateral approaches) (Pala et al., 2024), while one study did not report the surgical approach used (Xiao et al., 2025). In terms of patient allocation: a total of 1,296 DFNF cases were treated with DMC-THA, and 1,208 DFNF cases were in the BHA group. The selection process is shown in Fig. 1.

**Table 1:** The characteristics of included studies in the meta-analysis.

First author (year)	Country	Type	Age, mean, year (SD)	Gender (F/M)	Femoral neck fracture (*n*)	Surgical approach	Follow-up, mon (SD)	Fixation (cementless/cemented, *n*)	
								Cup	Stem
Xiao et al. (2025)	China	Retrospective cohort study	DMC group: 68.7 (±4.6)	DMC group: 59/43	DMC group: 102	NR	DMC group: 12 (NR)	DMC group: 0/102	DMC group: 0/102
			BHA group: 69.1 (±5.3)	BHA group: 52/42	BHA group: 94		BHA group: 12 (NR)	BHA group: N/A	BHA group: 0/94
Pala et al. (2024)	Italy	Retrospective cohort study	DMC group: 74 (±7.5)	DMC group: 61/19	DMC group: 80	26 Anterior,13 Posterolateral	DMC group: 14.5 (NR)	DMC group: NR	DMC group: 66/22
			BHA group: 78 (±6)	BHA group: 20/62	BHA group: 82	123 Anterolateral and Lateral	BHA group: 10.3 (NR)	BHA group: N/A	BHA group: 0/82
Zakaria et al. (2023)	Egypt	Randomized controlled trial	DMC group: 68.95 (±4.57)	DMC group: 15/5	DMC group: 20	All patients received direct lateral	DMC group: 24 (NR)	DMC group: 12/8	DMC group: 20/0
			BHA group: 72.50 (±7.03)	BHA group: 11/9	BHA group: 20		BHA group: 24 (NR)	BHA group: N/A	BHA group: 20/0
Rotini et al. (2022)	Italy	Retrospective cohort study	DMC group: 84.78 (NR)	Overall: 86 males, 214 females	DMC group: 209	All patients received posterolateral	DMC group: six (NR)	DMC group: NR	DMC group: NR
			BHA group: 87.03 (NR)		BHA group: 93		BHA group: six (NR)	BHA group: N/A	BHA group: NR
Valcarenghi et al. (2022)	Belgium	Retrospective cohort study	DMC group: 81 (±9)	DMC group: 97/21	DMC group: 118	All patients received posterolateral	DMC group: 18.1 (±7)	DMC group: 0/118	DMC group: 0/118
			BHA group: 85 (±8)	BHA group: 113/26	BHA group: 139		BHA group: 17.6 (±7)	BHA group: N/A	BHA group: 0/139
Bertault-Le Gourrierec et al. (2021)	France	Retrospective cohort study	DMC group: 78.5 (±9)	DMC group: 111/42	DMC group: 153	All patients received posterolateral	DMC group: 60 (NR)	DMC group: 12/141	DMC group: 23/130
			BHA group: 85.4 (±7.57)	BHA group: 84/28	BHA group: 112		BHA group: 60 (NR)	BHA group: N/A	BHA group: 84/28
Makeen et al. (2021)	Egypt	Randomized controlled trial	DMC group: 70.38 (±5.73)	DMC group: 9/7	DMC group: 16	All patients received posterolateral	DMC group: 24 (NR)	DMC group: 0/16	DMC group: 0/16
			BHA group: 71.12 (±6.28)	BHA group: 7/10	BHA group: 17		BHA group: 24 (NR)	BHA group: N/A	BHA group: NR
Nonne et al. (2019)	Italy	Retrospective cohort study	DMC group: 87.6 (±4)	DMC group: 45/15	DMC group: 60	All patients received posterolateral	DMC group: 28.3 (±4.75)	DMC group: 2/58	DMC group: 0/60
			BHA group: 86.1 (±6.25)	BHA group: 66/22	BHA group: 88		BHA group: 28.3 (±4.75)	BHA group: N/A	BHA group: 13/75
Ukaj et al. (2019)	Kosova	Prospective comparative study	DMC group: 78.11 (±5.40)	DMC group: 15/32	DMC group: 47	All patients received posterolateral	DMC group: 36 (NR)	DMC group: 0/47	DMC group: 31/16
			BHA group: 77.64 (±4.7)	BHA group: 24/23	BHA group: 47		BHA group: 36 (NR)	BHA group: N/A	BHA group: 0/47
Fahad et al. (2019)	Pakistan	Retrospective cohort study	DMC group: 69.3 (±9)	DMC group: 14/13	DMC group: 27	All patients received posterolateral	DMC group: 19 (±5.4)	DMC group: 0/27	DMC group: 0/27
			BHA group: 71.1 (±10.9)	BHA group: 46/31	BHA group: 77		BHA group: 20.6 (±6.6)	BHA group: N/A	BHA group: 0/77
Ochi et al. (2017)	Japan	Retrospective cohort study	DMC group: 80.0 (±7.9)	DMC group: 26/7	DMC group: 33	All patients received direct lateral	DMC group: 15.8 (±10.6)	DMC group: 0/33	DMC group: 0/33
			BHA group: 75.4 (±7.9)	BHA group: 16/4	BHA group: 20		BHA group: 28.2 (±26.6)	BHA group: N/A	BHA group: 0/20
Iorio et al. (2019)	Italy	Randomized controlled trial	DMC group: 82 (±4)	DMC group: 18/12	DMC group: 30	All patients received posterolateral	DMC group: 12 (NR)	DMC group: 0/30	DMC group: 0/30
			BHA group: 83 (±3)	BHA group: 17/13	BHA group: 30		BHA group: 12 (NR)	BHA group: N/A	BHA group: 0/30
Zagorov et al. (2018)	Bulgaria	Retrospective cohort study	DMC group: 73.4 (±12.5)	DMC group: 30/12	DMC group: 44	All patients received posterolateral	DMC group: 29.7 (±13.67)	DMC group: 3/41	DMC group: 22/19
			BHA group: 83.5 (±6.75)	BHA group: 26/6	BHA group: 32		BHA group: 23.1 (±14.13)	BHA group: N/A	BHA group: 0/32
Boukebous et al. (2018)	France	Retrospective cohort study	DMC group: 77.8 (±10.8)	DMC group: 70/28	DMC group: 98	All patients received posterolateral	DMC group: 24.2 (NR)	DMC group: 29/69	DMC group: 44/54
			BHA group: 83.3 (±8.9)	BHA group: 73/28	BHA group: 101		BHA group: 25.8 (NR)	BHA group: N/A	BHA group: 79/22
Kim et al. (2018)	South Korea	Retrospective cohort study	DMC group: 73.1 (±6.0)	DMC group: 58/26	DMC group: 84	All patients received posterolateral	DMC group: 21.7 (±10.4)	DMC group: 0/84	DMC group: 0/84
			BHA group: 72.9 (±7.8)	BHA group: 57/27	BHA group: 84		BHA group: 22.1 (±9.6)	BHA group: N/A	BHA group: 0/84
Bensen, Jakobsen & Krarup (2014)	Denmark	Retrospective cohort study	DMC group: 75.2 (NR)	DMC group: 123/52	DMC group: 175	All patients received posterolateral	DMC group: 21.7 (NR)	DMC group: 3/172	DMC group: 9/166
			BHA group: 84.1 (NR)	BHA group: 131/40	BHA group: 171		BHA group: 25.3 (NR)	BHA group: N/A	BHA group: 9/162

**Notes:**

Values in “Fixation (cementless/cemented, *n*)” are presented as [number of cementless fixation cases]/[number of cemented fixation cases].

NR, Not reported; N/A, Not applicable.

### Quality of the included studies

Our research consisted of 13 non-RCTs (Pala et al., 2024; Kim et al., 2018; Bensen, Jakobsen & Krarup, 2014; Valcarenghi et al., 2022; Ochi et al., 2017; Xiao et al., 2025; Rotini et al., 2022; Zagorov et al., 2018; Nonne et al., 2019; Ukaj et al., 2019; Boukebous et al., 2018; Fahad et al., 2019; Bertault-Le Gourrierec et al., 2021), three RCTs (Zakaria et al., 2023; Makeen et al., 2021; Iorio et al., 2019). The quality of non-RCTs was shown in Table 2. In terms of Downs and Blacks score, all included studies were over 13. In terms of NOS, seven of eight non-RCTs had scored ≥6*. The lowest score was 5* due to low comparability and exposure (Fahad et al., 2019). The quality of RCTs is presented in Table 3. Of the three included RCTs, two were rated as high quality, and one was rated as moderate quality. Regarding methodological details: the randomization methods, similar baseline and patient compliance were explicitly described in these studies. However, none of the studies provided care provider blinded. The value of weighted kappa for the agreement on these studies between reviewers was excellent (K = 0.78).

**Table 2:** Quality of non-RCTs.

Author (year)	Country	Type	Study quality				
Downs and black score				NOS			
				Selection	Comparability	Outcome	Total score
Xiao et al. (2025)	China	Retrospective cohort study	17	****	**	**	*******
Pala et al. (2024)	Italy	Retrospective cohort study	18	****	**	***	*********
Rotini et al. (2022)	Italy	Retrospective cohort study	16	***	*	**	******
Valcarenghi et al. (2022)	Belgium	Retrospective cohort study	17	****	**	**	********
Bertault-Le Gourrierec et al. (2021)	France	Retrospective cohort study	18	****	**	***	*********
Nonne et al. (2019)	Italy	Retrospective cohort study	16	***	*	**	******
Ukaj et al. (2019)	Kosova	Prospective comparative study	15	***	*	**	******
Fahad et al. (2019)	Pakistan	Retrospective cohort study	16	**	*	**	*****
Zagorov et al. (2018)	Bulgaria	Retrospective cohort study	16	***	*	***	*******
Boukebous et al. (2018)	France	Retrospective cohort study	16	***	*	**	******
Kim et al. (2018)	South Korea	Retrospective cohort study	18	****	*	***	********
Ochi et al. (2017)	Japan	Retrospective cohort study	15	***	*	**	******
Bensen, Jakobsen & Krarup (2014)	Denmark	Retrospective cohort study	17	***	**	**	*******

**Note:**

Each asterisk (*) represents one point for the corresponding quality item.

**Table 3:** Quality of RCTs.

RCTs	Randomized adequately^a^	Allocation concealed	Patient blinded	Care provider blinded	Outcome assessor blinded	Acceptable drop-out rate^b^	ITT analysis^c^	Avoided selective reporting	Similar baseline	Similar or avoided cofactor	Patient compliance	Similar timing	Quality^d^
Zakaria et al. (2023)	Yes	Unclear	Yes	Unclear	Unclear	Yes	Unclear	Yes	Yes	Yes	Yes	Yes	High
Makeen et al. (2021)	Yes	Unclear	Unclear	Unclear	Unclear	No	Unclear	Yes	Yes	Yes	Yes	Yes	Moderate
Iorio et al. (2019)	Yes	Yes	Yes	Unclear	Yes	No	Yes	No	Yes	Unclear	Yes	Yes	High

**Notes:**

a Only if the method of sequence made was explicitly introduced could get a ‘Yes’.

b Drop-out rate <20% could get a ‘Yes’, otherwise ‘No’.

c ITT = intention-to-treat, only if all randomized participants were analyzed in the group they were allocated to could receive a ‘Yes’.

d “Yes” items more than 7 means ‘High’; more than 4 but no more than 7 means ‘Moderate’; no more than 4 means ‘Low’.

Unclear = Corresponding methodological details not reported in original studies.

Abbreviations: RCT, Randomized controlled trial; ITT, Intention-to-treat analysis; SAE, Serious adverse event.

### Clinical outcomes

#### Dislocation rate

**Figure 2:**
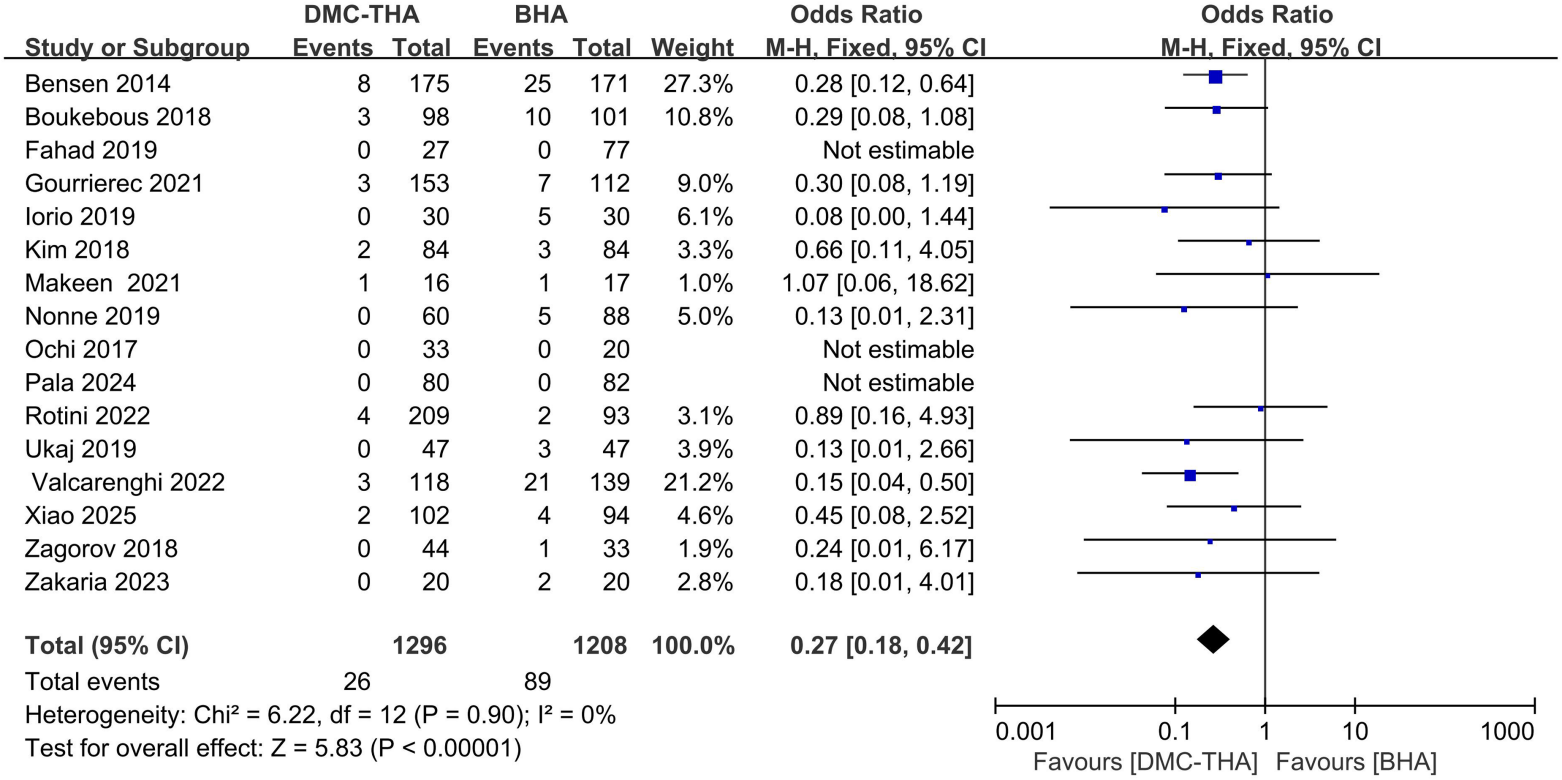
The proportion of dislocation rate between DMC-THA and BHA.

Dislocation rate was reported in 16 studies (Pala et al., 2024; Kim et al., 2018; Bensen, Jakobsen & Krarup, 2014; Valcarenghi et al., 2022; Zakaria et al., 2023; Ochi et al., 2017; Xiao et al., 2025; Rotini et al., 2022; Zagorov et al., 2018; Nonne et al., 2019; Ukaj et al., 2019; Boukebous et al., 2018; Fahad et al., 2019; Bertault-Le Gourrierec et al., 2021; Makeen et al., 2021; Iorio et al., 2019), with low heterogeneity observed (*P* = 0.90; I^2^ = 0%). There were 26 patients of 1,296 (2.01%) in the DMC-THA group and 89 patients of 1,208 (7.37%) in the BHA group. Patients undergoing DMC-THA had significantly lower dislocation rate compared with those undergoing BHA (OR: 0.27; 95% CI [0.18–0.42]; *P* < 0.00001, Fig. 2).

#### Reoperation rate

**Figure 3:**
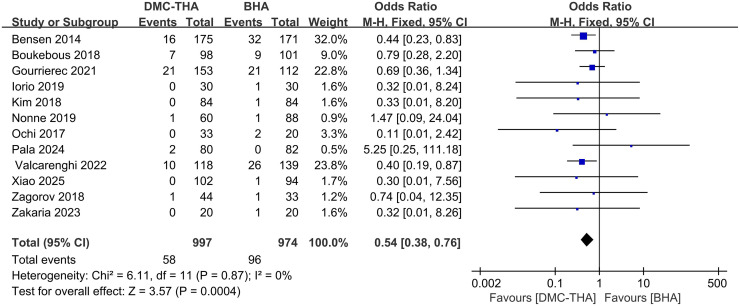
The proportion of reoperation rate between DMC-THA and BHA.

Reoperation rate was reported in 12 studies (Pala et al., 2024; Kim et al., 2018; Bensen, Jakobsen & Krarup, 2014; Valcarenghi et al., 2022; Zakaria et al., 2023; Ochi et al., 2017; Xiao et al., 2025; Zagorov et al., 2018; Nonne et al., 2019; Boukebous et al., 2018; Bertault-Le Gourrierec et al., 2021; Iorio et al., 2019), with low heterogeneity observed (*P* = 0.87; I^2^ = 0%). There were 58 patients of 997 (5.82%) in the DMC-THA group and 96 patients of 974 (9.86%) in the BHA group. The majority studies were performed with non-cemented fixation. A significant decrease in reoperation rate was observed in patients undergoing DMC-THA compared with those undergoing BHA (OR: 0.54; 95% CI [0.38–0.76]; *P* = 0.0004, Fig. 3).

### Mortality

**Figure 4:**
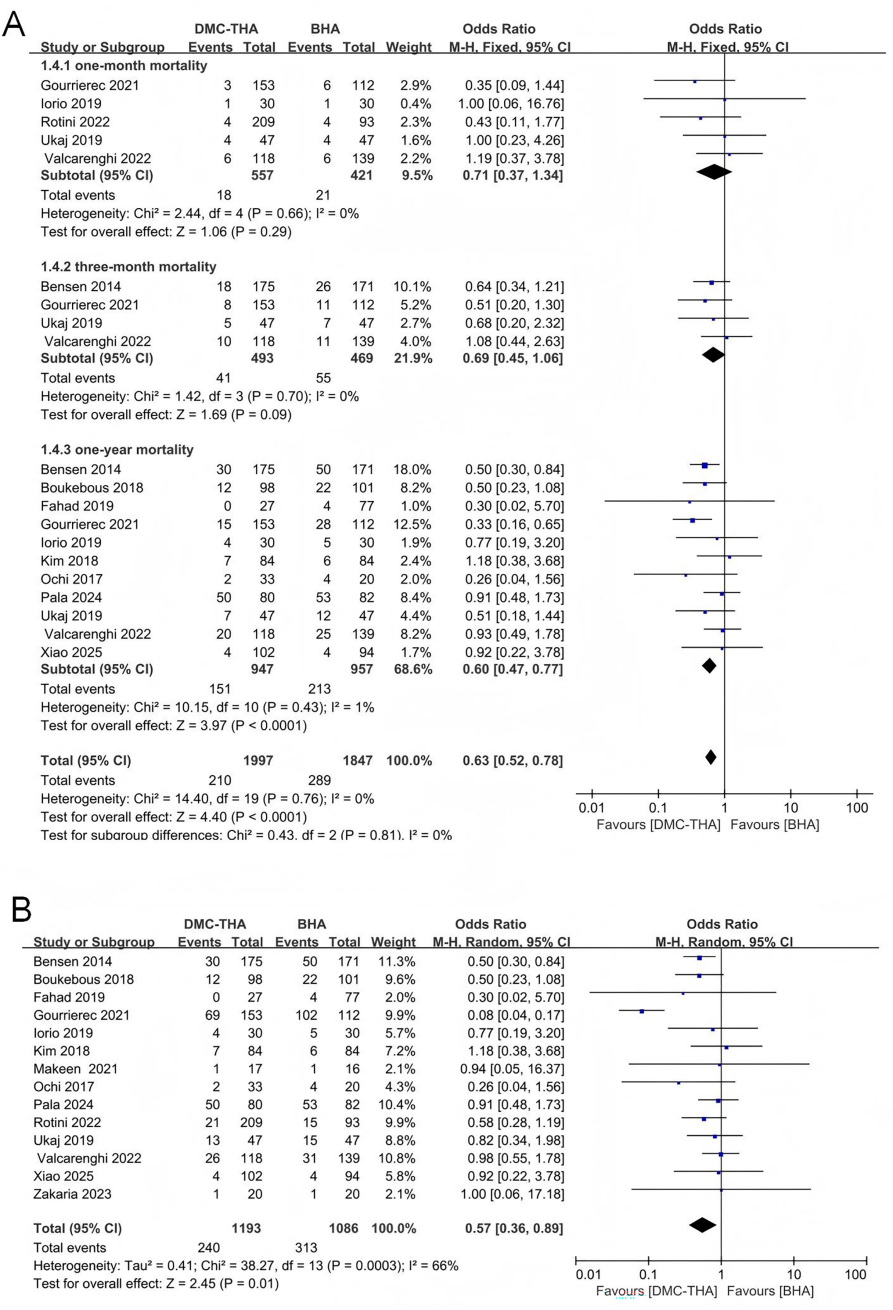
(A) One-month, 3-month and 1-year mortality difference between DMC-THA and BHA. (B) Last follow-up mortality difference between DMC-THA and BHA.

One-month mortality was reported in five studies (Valcarenghi et al., 2022; Rotini et al., 2022; Ukaj et al., 2019; Bertault-Le Gourrierec et al., 2021; Iorio et al., 2019), with low heterogeneity observed (*P* = 0.66; I^2^ = 0%). There were 18 patients of 557 (3.23%) in the DMC-THA group and 21 patients of 421 (4.99%) in the BHA group. No significant decrease in 1-month mortality was observed in patients undergoing DMC-THA compared with those undergoing BHA (OR: 0.71; 95% CI [0.37–1.34]; *P* = 0.29, Fig. 4A).

Three-month mortality was reported in four studies (Bensen, Jakobsen & Krarup, 2014; Valcarenghi et al., 2022; Ukaj et al., 2019; Bertault-Le Gourrierec et al., 2021), with low heterogeneity observed (*P* = 0.70; I^2^ = 0%). There were 41 patients of 493 (8.32%) in the DMC-THA group and 55 patients of 469 (11.73%) in the BHA group. No significant decrease in 3 months was observed in patients undergoing DMC-THA compared with those undergoing BHA (OR: 0.69; 95% CI [0.45–1.06]; *P* = 0.09, Fig. 4A).

One-year mortality was reported in 11 studies (Pala et al., 2024; Kim et al., 2018; Bensen, Jakobsen & Krarup, 2014; Valcarenghi et al., 2022; Ochi et al., 2017; Xiao et al., 2025; Ukaj et al., 2019; Boukebous et al., 2018; Fahad et al., 2019; Bertault-Le Gourrierec et al., 2021; Iorio et al., 2019), with low heterogeneity observed (*P* = 0.43; I^2^ = 1.00%). There were 151 patients of 947 (15.95%) in the DMC-THA group and 213 patients of 957 (22.26%) in the BHA group. DMC-THA was associated with a significantly lower 1-year mortality compared with BHA (OR: 0.60; 95% CI [0.47–0.77]; *P* < 0.0001, Fig. 4A), though this result should be interpreted with caution given the non-randomized study design and potential selection bias.

Last follow-up mortality was reported in 15 studies (Pala et al., 2024; Kim et al., 2018; Bensen, Jakobsen & Krarup, 2014; Valcarenghi et al., 2022; Zakaria et al., 2023; Ochi et al., 2017; Xiao et al., 2025; Rotini et al., 2022; Zagorov et al., 2018; Nonne et al., 2019; Ukaj et al., 2019; Boukebous et al., 2018; Fahad et al., 2019; Bertault-Le Gourrierec et al., 2021; Iorio et al., 2019), with moderate heterogeneity observed (*P* = 0.0003; I^2^ = 66%). Therefore, the random-effects model was used. There were 240 patients of 1,193 (20.12%) in the DMC-THA group and 313 patients of 1,086 (28.82%) in the BHA group. DMC-THA was associated with a significantly lower mortality at final follow-up compared with BHA (OR: 0.57; 95% CI [0.36–0.89]; *P* = 0.01, Fig. 4B). However, this finding should be interpreted cautiously given the non-randomized study design and potential selection bias.

### Operative time

**Figure 5:**
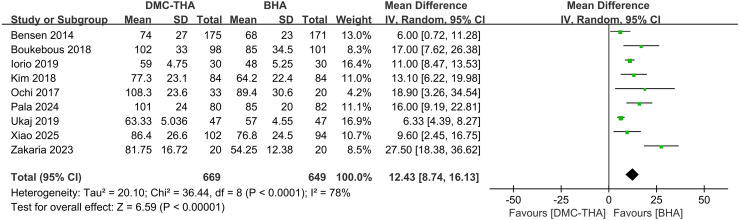
Operative time between DMC-THA and BHA.

Operative time was presented in nine studies (Pala et al., 2024; Kim et al., 2018; Bensen, Jakobsen & Krarup, 2014; Zakaria et al., 2023; Ochi et al., 2017; Xiao et al., 2025; Ukaj et al., 2019; Boukebous et al., 2018; Iorio et al., 2019), with a relatively high heterogeneity observed (*P* < 0.0001; I^2^ = 78%). Therefore, the random-effects model was used. Operative time was significantly longer in DMC-THA than in the BHA group (MD: 12.43; 95% CI [8.74–16.13]; *P* < 0.00001, Fig. 5).

### Intraoperative blood loss

Intraoperative blood loss was reported in six studies (Kim et al., 2018; Bensen, Jakobsen & Krarup, 2014; Zakaria et al., 2023; Ochi et al., 2017; Xiao et al., 2025; Ukaj et al., 2019). Substantial heterogeneity was observed (*P* < 0.00001; I^2^ = 91%); therefore, a meta-analysis was not performed for this outcome. All studies reported that intraoperative blood loss was significantly greater in the DMC-THA group than in the BHA group.

### Transfusion rate

**Figure 6:**
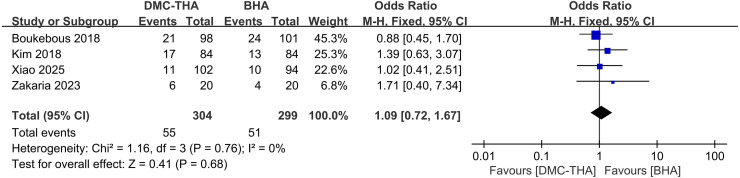
The proportion of transfusion rate between DMC-THA and BHA.

Four studies reported transfusion rate, low heterogeneity was observed in both groups (*P* = 0.76; I^2^ = 0%) (Kim et al., 2018; Zakaria et al., 2023; Xiao et al., 2025; Boukebous et al., 2018). There were 55 patients of 304 (18.09%) in the DMC-THA group and 51 patients of 299 (17.06%) in the BHA group. No significant difference was observed in transfusion rate between patients undergoing DMC-THA compared with those undergoing BHA (OR: 1.09; 95% CI [0.72–1.67]; *P* = 0.68, Fig. 6).

### Infection rate

**Figure 7:**
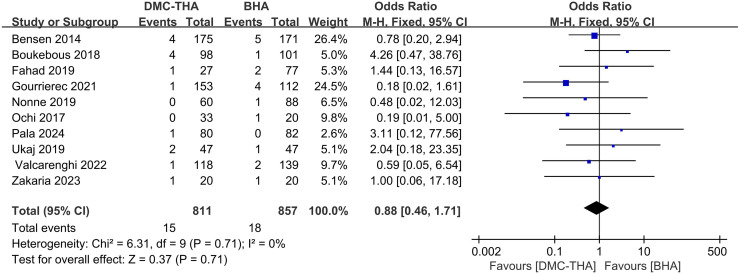
The proportion of infection rate between DMC-THA and BHA.

Ten studies reported infection rate, low heterogeneity was observed in both groups (*P* = 0.71; I^2^ = 0%) (Pala et al., 2024; Bensen, Jakobsen & Krarup, 2014; Valcarenghi et al., 2022; Zakaria et al., 2023; Ochi et al., 2017; Nonne et al., 2019; Ukaj et al., 2019; Boukebous et al., 2018; Fahad et al., 2019; Bertault-Le Gourrierec et al., 2021). There were 15 patients of 811 (1.85%) in the DMC-THA group and 18 patients of 857 (2.10%) in the BHA group. No significant difference was observed in infection rate between patients undergoing DMC-THA compared with those undergoing BHA (OR: 0.88; 95% CI [0.46–1.71]; *P* = 0.71, Fig. 7).

### Fracture

**Figure 8:**
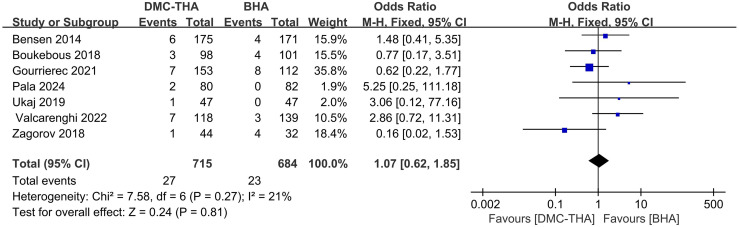
The proportion of fracture between DMC-THA and BHA.

Seven studies reported fracture, low heterogeneity was observed in both groups (*P* = 0.27; I^2^ = 21%) (Pala et al., 2024; Bensen, Jakobsen & Krarup, 2014; Valcarenghi et al., 2022; Zakaria et al., 2023; Ukaj et al., 2019; Boukebous et al., 2018; Bertault-Le Gourrierec et al., 2021). There were 27 patients of 715 (3.78%) in the DMC-THA group and 23 patients of 684 (3.36%) in the BHA group. No significant difference was observed in postoperative fracture between patients undergoing DMC-THA compared with those undergoing BHA (OR: 1.07, 95% CI [0.62–1.85]; *P* = 0.81, Fig. 8).

### Loosening of components

**Figure 9:**
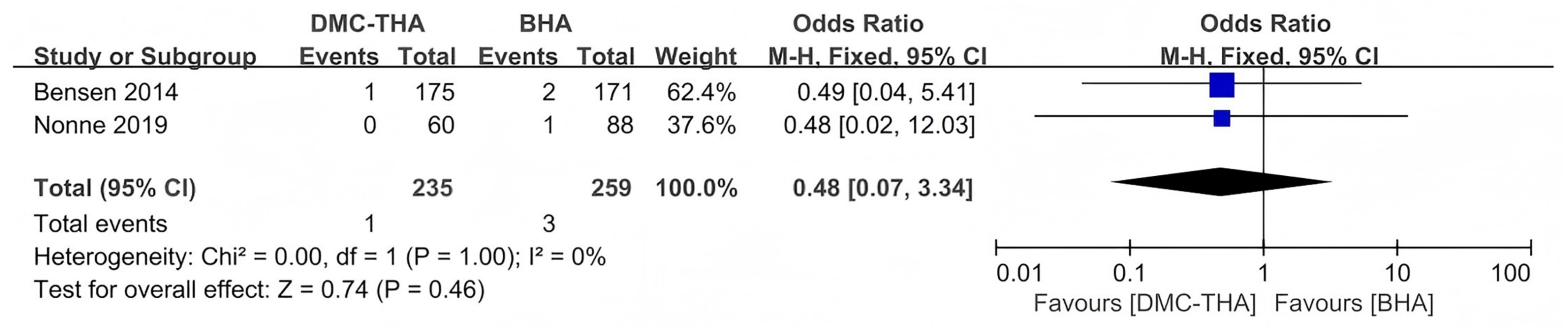
The proportion of loosening of components between DMC-THA and BHA.

Two studies reported loosening of components, low heterogeneity was observed in both groups (*P* = 1.00; I^2^ = 0%) (Bensen, Jakobsen & Krarup, 2014; Nonne et al., 2019). There was one patient of 235 (0.43%) in the DMC-THA group and three patients of 259 (1.16%) in the BHA group. No significant difference was observed in loosening of components between patients undergoing DMC-THA compared with those undergoing BHA (OR: 0.48, 95% CI [0.07–3.34]; *P* = 0.46, Fig. 9).

### Thromboembolism

**Figure 10:**
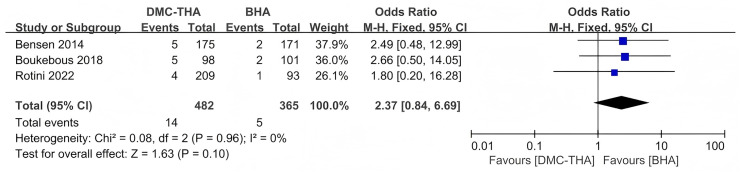
The proportion of thromboembolism between DMC-THA and BHA.

Three studies reported thromboembolism. Low heterogeneity was observed in both groups (*P* = 0.96; I^2^ = 0%) (Bensen, Jakobsen & Krarup, 2014; Rotini et al., 2022; Boukebous et al., 2018). There were 14 patients of 482 (2.90%) in the DMC-THA group and five patients of 365 (1.37%) in the BHA group. No significant difference was observed in thromboembolism between patients undergoing DMC-THA compared with those undergoing BHA (OR: 2.37, 95% CI [0.84–6.69]; *P* = 0.10, Fig. 10).

### Heterotopic ossification

**Figure 11:**
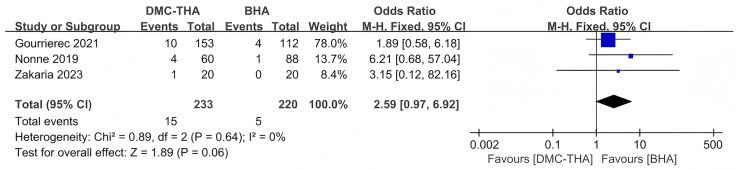
The proportion of heterotopic ossification between DMC-THA and BHA.

Three studies reported heterotopic ossification, low heterogeneity was observed in both groups (*P* = 0.64; I^2^ = 0%) (Zakaria et al., 2023; Nonne et al., 2019; Bertault-Le Gourrierec et al., 2021). There were 15 patients of 233 (6.44%) in the DMC-THA group and five patients of 220 (2.27%) in the BHA group. No significant difference was observed in postoperative heterotopic ossification between patients undergoing DMC-THA compared with those undergoing BHA (OR: 2.59, 95% CI [0.97–6.92]; *P* = 0.06, Fig. 11).

### HHS

**Figure 12:**
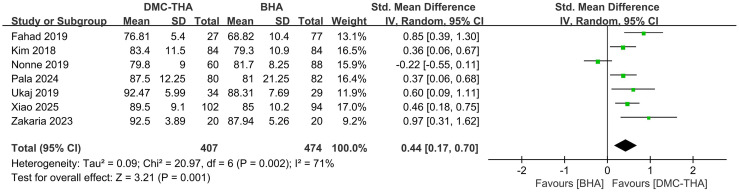
The proportion of HHS between DMC-THA and BHA.

HHS was reported in seven studies (Pala et al., 2024; Kim et al., 2018; Zakaria et al., 2023; Xiao et al., 2025; Nonne et al., 2019; Ukaj et al., 2019; Fahad et al., 2019). Significant heterogeneity was observed among these studies (*P* = 0.002; I^2^ = 71%), partly attributed to inconsistent follow-up time points across studies. To avoid bias in pooled results caused by this temporal heterogeneity, the standardized mean difference (SMD) was used for data pooling under a random-effects model. The pooled results indicated that DMC-THA was associated with significantly higher Harris Hip Scores than BHA (SMD = 0.44, 95% CI [0.17–0.70], *P* = 0.001; Fig. 12).

### Hospitalization period

**Figure 13:**
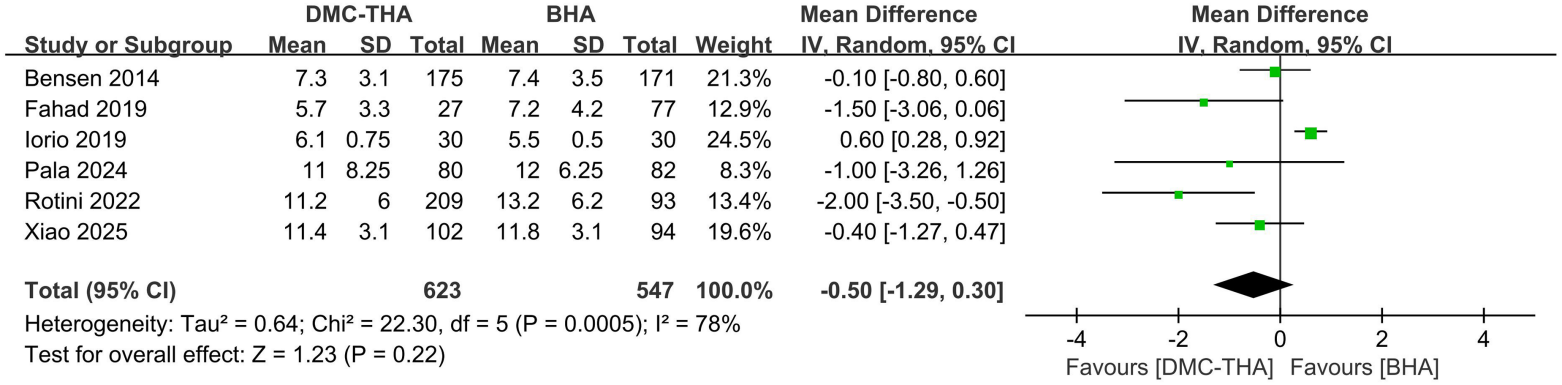
The proportion of hospitalization period between DMC-THA and BHA.

Hospitalization period was reported in six studies (Pala et al., 2024; Bensen, Jakobsen & Krarup, 2014; Xiao et al., 2025; Rotini et al., 2022; Fahad et al., 2019; Iorio et al., 2019). There was significant heterogeneity between the studies (*P* = 0.0005; I^2^ = 78%); therefore, the random-effects model was used. No significant difference was found between the two groups in hospitalization period (MD = −0.50, 95%CI [−1.29 to 0.30], *P* = 0.22; Fig. 13).

### Publication bias

**Figure 14:**
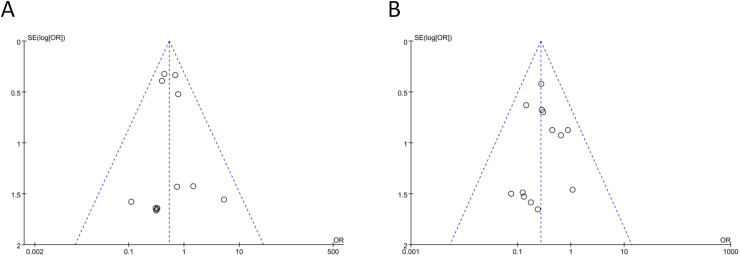
(A) Publication bias of dislocation rate. (B) Publication bias of reoperation rate.

Funnel plots were constructed to assess for potential publication bias in the analyses of dislocation rate (Fig. 14A) and reoperation rate (Fig. 14B). Visual inspection revealed no marked asymmetry in either plot, indicating no significant qualitative evidence of publication bias in the current meta-analysis.

### Sensitivity analyses

One study was individually deleted each time to observe its influence on the pooled SMD, MD or OR. The results showed that no study could substantially affect the pooled SMD, MD or OR in present meta-analysis.

## Discussion

Our meta-analysis demonstrated that the use of DMC-THA was associated with significantly decreased rates of dislocation, reoperation, 1-year mortality, and final follow-up mortality, as well as improved HHS scores. Though longer operative time was observed in DMC-THA, no significant differences were found in 1-month mortality, 3-month mortality, transfusion rate, infection rate, fracture, loosening of components, thromboembolism, heterotopic ossification, and hospitalization period between the two groups.

Dislocation is a common complication and the leading cause of revision following hip arthroplasty (Ukaj et al., 2019). Although THA with a conventional cup was associated with better clinical outcomes and lower risk of revision, it had a higher dislocation rate after treatment for DFNFs compared with BHA (Bensen, Jakobsen & Krarup, 2014; Levin et al., 2018). Thus, BHA is regarded as a preferred treatment for patients with DFNFs. DMC was initially designed to reduce the rate of dislocation when using THA to treat DFNFs, and it has been reported with a lower dislocation rate than conventional THA (Hwang et al., 2018). In our study, the DMC-THA group had significantly lower rate of dislocation (2.01% *vs*. 7.37%) compared with the BHA group. Ukaj et al. (2019) also found DMC was beneficial to THA in terms of dislocation rate with a minimum 3-year follow-up. For non-cooperative patients, DMC-THA may be a safe and reliable choice, as these patients are at higher risk of dislocation due to uncontrolled movements and falls. Iorio et al. (2019) reported the DMC-THA group had significantly reduced dislocation rate at 1 year after operation in patients with dementia. Surgical approach is also an important factor that influences dislocation rate (Kim et al., 2025). Currently, the best surgical approach in the treatment of DFNFs is still controversial. The main surgical approaches include posterolateral and direct anterior. Direct anterior approach (DAA) had been proven with lower dislocation rate compared to the conventional THA (Meermans et al., 2017; Wang et al., 2018). But the main limitations of DAA are the high incidence of intraoperative femur fracture and postoperative periprosthetic fracture (Kong et al., 2019). In addition, Kristensen et al. (2016) found the patients with posterolateral approach had better outcomes in functional outcomes, pain and patient satisfaction compared to the patients with an anterior approach (Homma et al., 2016). However, the effect of surgical approach on dislocation does not seem to apply to DMC-THA. Boukebous et al. (2018) found that the dislocation rate of DMC-THA with posterolateral approach was comparable to that of anterior approach.

The meta-analysis showed that the reoperation rate of DMC-THA group was significantly lower than that of BHA group (5.82% *vs*. 9.86%). In Bensen’s study with 346 patients, they found the DMC-THA was superior to BHA in terms of reoperation rate after DFNFs treatment (9.1% *vs*. 18.71%) (Bensen, Jakobsen & Krarup, 2014). The common causes of reoperation include dislocation, loosening of components, deep infection, and post-operative periprosthetic fracture, among which dislocation is still the main cause of reoperation (Yeroushalmi et al., 2023). In addition, acetabulum erosion is also a frequent cause that results in a higher reoperation rate after HA (Bertault-Le Gourrierec et al., 2021). This is a time-dependent process, long-term prospective studies are necessary to evaluate the effect.

The meta-analysis demonstrated that DMC-THA showed no significant difference in 1-month mortality (3.23% *vs*. 4.99%) and 3-month mortality (8.32% *vs*. 11.73%), but was associated with a significantly lower 1-year mortality (15.95% *vs*. 22.26%) and a lower mortality (12.55% *vs*. 19.43%) at the last follow-up (20.12% *vs*. 28.82%). It should be noted that this finding is limited by confounding by indication, as healthier patients were more likely to receive DMC-THA. As the majority of included studies are observational, unmeasured confounding factors—such as pre-fracture functional status, cognitive function, and social support—may influence survival outcomes. However, a plausible explanation for this observation could be the superior functional recovery and lower reoperation rates associated with DMC-THA, which may contribute to earlier mobilization and reduced complications in the fragile elderly population. Bertault-Le Gourrierec et al. (2021) found through a 5-year follow-up study that the overall survival of patients in the BHA group (median = 29 months) was significantly shorter than that in the DMC-THA group (median = 94 months).

Our meta-analysis showed that DMC-THA was associated with a longer length of surgery. It could be attributed to more complicated and technical procedures using DMC (Kim et al., 2025). The longer operation time could lead to more perioperative blood loss (Rotini et al., 2022). Previous studies have reported THA with traditional cup was associated higher rates of complications. Some studies reported the use of DMC-THA was associated with more perioperative blood loss compared with BHA (Kim et al., 2018; Bensen, Jakobsen & Krarup, 2014; Zakaria et al., 2023; Ochi et al., 2017; Xiao et al., 2025; Ukaj et al., 2019). This finding raised concerns about the effect on transfusion rate. So, we assessed the potential risk of the longer procedure in these indicators: transfusion rate, infection rate, fracture, loosening of components, thromboembolism, and heterotopic ossification. However, no significant differences were found in complication rate between the two groups. Xiao et al. (2025) have reported that DMC-THA is associated with a significant reduction in blood loss, though no differences were found in postoperative drainage volume or perioperative blood transfusion rates. Ukaj et al. (2019) also reported similar short-term complications between them. However, it has been reported that DMC-THA had a good long-term effect on osteoarthritis and avascular necrosis of the femoral head, and a lower rate of complications was found, especially in patients over 70 years old (Zagorov et al., 2018). Therefore, good long-term results with DMC-THA can also be expected in the treatment of DFNFs. Regarding the functional score (HHS), it is one of the signs of successful operation. Previous studies have outlined THA leads to better functional results than BHA (Epinette et al., 2016; Bouche et al., 2020). In our research, six of seven studies reported that patients undergoing DMC-THA had significantly better functional outcomes, as indicated by higher HHS scores, compared to those undergoing BHA (Pala et al., 2024; Kim et al., 2018; Zakaria et al., 2023; Xiao et al., 2025; Nonne et al., 2019; Ukaj et al., 2019; Fahad et al., 2019). During a minimum follow-up period of 5 years, Bertault-Le Gourrierec et al. (2021) evaluated treatment outcomes using the WOMAC (Western Ontario and McMaster Universities Osteoarthritis Index) score. Their findings showed that the treatment outcomes in the DMC group were significantly superior to those in the BHA group.

Furthermore, the concern about the prolongation of the hospitalization period did not materialize, and there was no significant difference in the length of hospital stay between the two groups of patients. Rotini further grouped the patients by age, and no significant statistical differences were observed among patients of different age groups (Heshmat et al., 2024).

### Aspects requiring attention in future research

(1) High-quality randomized controlled trials with more uniformity in outcomes reporting are desirable for further research. (2) Differences in dislocation rate, reoperation needs, and mortality must be evaluated in long-term follow-up. Further research is needed to assess long-term results. (3) Some important clinical parameters (limb length) and radiological parameters (implant orientation, mechanical axis, acetabular protrusion) should be recorded and analyzed. (4) Though DMC-THA has been reported to have better cost-effectiveness than conventional THA (Bouche et al., 2020), further studies are required to compare the hospital and socioeconomic cost-effectiveness between DMC-THA and BHA.

### Limitations identified with this study

(1) Selection bias and confounding by indication: The majority of included studies are observational. There is a potential for “healthy cohort” bias, where DMC-THA might be preferentially selected for fitter patients with better life expectancy, while BHA is reserved for frailest patients. This imbalance in baseline health status may confound the observed survival benefit of DMC-THA. (2) Few studies had sufficient postoperative follow-up duration, which limited us to obtain more rigorous and convincing outcomes. (3) Only three RCTs were included, which may influence the accuracy of the pooled results. (4) High heterogeneity of some results was found in this meta-analysis. The age, surgical approach, type of implant fixation, surgical techniques, type of component and follow-up time were considered as the reasons. But we cannot perform subgroup analysis by these factors due to a lack of data. (5) Publication bias cannot be completely excluded. Although visual inspection of funnel plots for dislocation and reoperation rates revealed no significant qualitative evidence of publication bias, the inherent limitations of visual assessment and the potential for unpublished negative studies preclude a definitive conclusion.

## Conclusion

DMC-THA may be associated with lower rates of dislocation, reoperation, and mortality compared with BHA in the treatment of DFNFs. However, given the predominantly observational nature of the included studies, these findings do not establish a definitive causal relationship. While DMC-THA is associated with a longer operative time, it does not appear to increase the risk of complications such as transfusion, infection, fracture, or thromboembolism. However, the evidence supporting these findings remains limited, and DMC-THA should be considered a potential treatment option rather than a routinely recommended approach for DFNFs. More high-quality studies with large sample sizes, adequate follow-up periods, and standardized outcome reporting are needed to further validate the long-term efficacy and safety of DMC-THA relative to BHA.

## Supplemental Information

10.7717/peerj.21535/supp-1 Supplemental Information 1 Raw Data Extracted from Included Studies for Meta-Analysis.

10.7717/peerj.21535/supp-2 Supplemental Information 2 PRISMA checklist.

10.7717/peerj.21535/supp-3 Supplemental Information 3 Search Strings.

10.7717/peerj.21535/supp-4 Supplemental Information 4 Target Audience.

## List of abbreviations

THA: Total Hip Arthroplasty
BHA: Bipolar Hemiarthroplasty
DFNFs: Displaced Femoral Neck Fractures
DMC: Dual Mobility Cup
DVT: Deep Vein Thrombosis
PE: Pulmonary Embolism
HHS: Harris Hip Score
RCTs: Randomized Controlled Trials
NOS: Newcastle-Ottawa Scale
DAA: Direct Anterior Approach
CI: Confidence Interval
OR: Odds Ratio
MD: Mean Difference

## Additional Information and Declarations

### Data Availability

The following information was supplied regarding data availability:

This is a Systematic review/Meta-analysis.
