# Supplementary figures and images for: Dual mobility total hip arthroplasty vs. bipolar hemiarthroplasty in treating patients with displaced femoral neck fractures: a systematic review and meta-analysis
Source: PeerJ. 2026 Jul 16;14:e21535. doi: 10.7717/peerj.21535 (PMC13380883; doi:10.7717/peerj.21535)

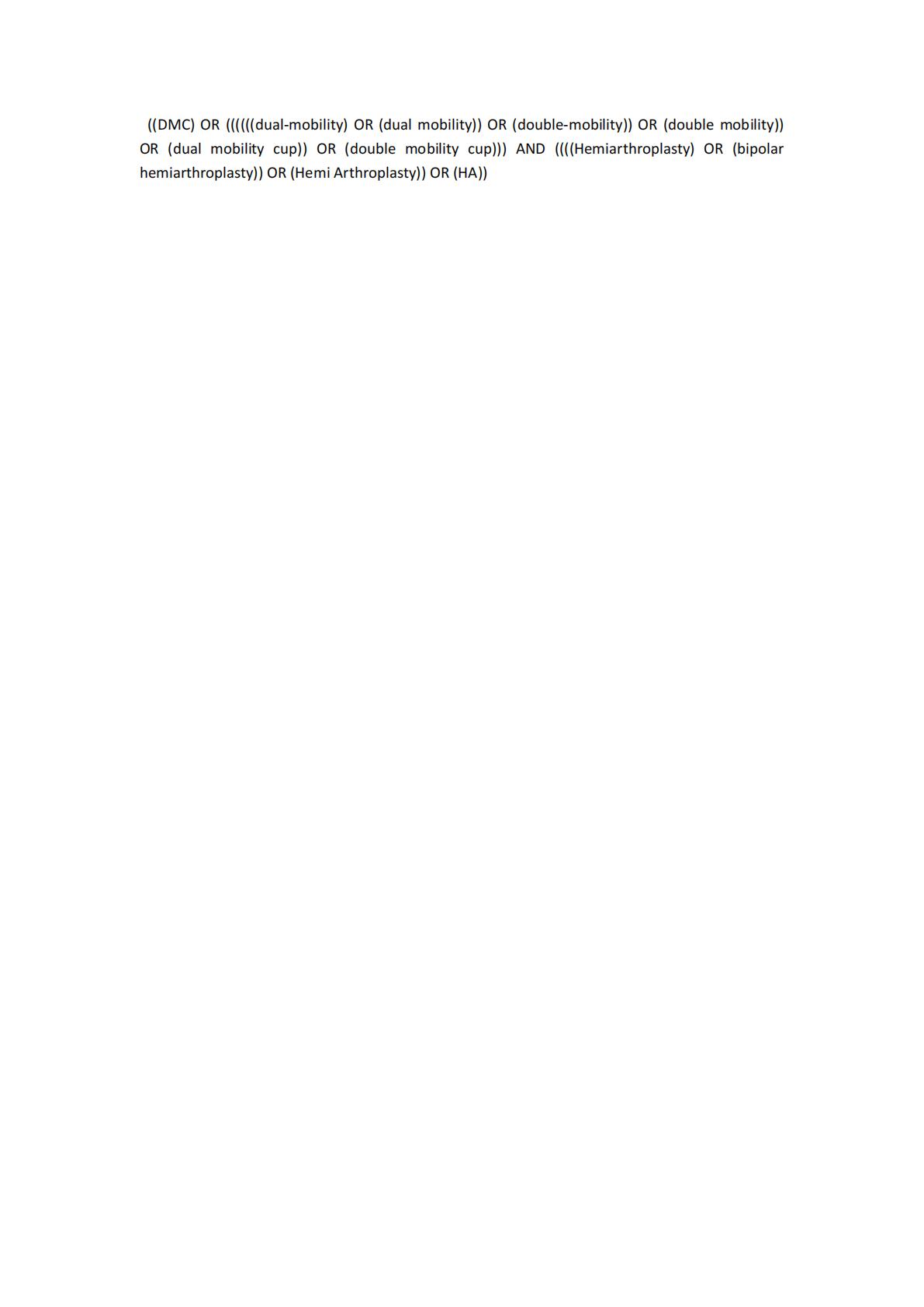

Supplement: Supplemental Information 3 [file peerj-14-21535-s003.jpg]
