# Supplementary material for: Dual mobility total hip arthroplasty vs. bipolar hemiarthroplasty in treating patients with displaced femoral neck fractures: a systematic review and meta-analysis
Source: PeerJ. 2026 Jul 16;14:e21535. doi: 10.7717/peerj.21535 (PMC13380883; doi:10.7717/peerj.21535)
Supplement: Supplemental Information 4 [file peerj-14-21535-s004.docx]

This systematic review and meta-analysis is intended for the following audiences:

1. ****Orthopedic and Joint Surgeons****: Clinicians involved in the management of displaced femoral neck fractures (DFNFs) in elderly or high-risk patients, who require evidence-based guidance to select optimal arthroplasty strategies (dual mobility total hip arthroplasty [DMC-THA] vs. bipolar hemiarthroplasty [BHA]).
2. ****Clinical Researchers****: Investigators focusing on orthopedic trauma, hip arthroplasty, or meta-analyses, who may use this study as a foundation for future high-quality trials (e.g., large-scale RCTs with long-term follow-up) or to identify research gaps (e.g., cost-effectiveness, radiological outcomes).
3. ****Healthcare Policy Makers and Hospital Administrators****: Stakeholders responsible for resource allocation, surgical protocol development, and formulary decisions for orthopedic implants, who need data on efficacy, safety, and long-term outcomes to optimize clinical pathways for DFNFs.
4. ****Geriatric Medicine Specialists****: Providers caring for elderly patients with DFNFs, who collaborate with orthopedic teams to balance early mobilization, complication prevention, and mortality reduction.

The study aims to address the clinical controversy surrounding DMC-THA vs. BHA for DFNFs, providing actionable evidence to inform shared decision-making between clinicians and patients.
